# Offering patients more: how the West Africa Ebola outbreak can shape innovation in therapeutic research for emerging and epidemic infections

**DOI:** 10.1098/rstb.2016.0294

**Published:** 2017-04-10

**Authors:** Amanda M. Rojek, Peter W. Horby

**Affiliations:** Epidemic Diseases Research Group Oxford (ERGO), Centre for Tropical Medicine and Global Health, Nuffield Department of Medicine, University of Oxford, Old Road Campus, Roosevelt Drive, Oxford OX3 7FZ, UK

**Keywords:** Ebola, epidemic, pandemic, clinical trial, viral haemorrhagic fever

## Abstract

Although, after an epidemic of over 28 000 cases, there are still no licensed treatments for Ebola virus disease (EVD), significant progress was made during the West Africa outbreak. The pace of pre-clinical development was exceptional and a number of therapeutic clinical trials were conducted in the face of considerable challenges. Given the on-going risk of emerging infectious disease outbreaks in an era of unprecedented population density, international travel and human impact on the environment it is pertinent to focus on improving the research and development landscape for treatments of emerging and epidemic-prone infections. This is especially the case since there are no licensed therapeutics for some of the diseases considered by the World Health Organization as most likely to cause severe outbreaks—including Middle East respiratory syndrome coronavirus, Marburg virus, Crimean Congo haemorrhagic fever and Nipah virus. EVD, therefore, provides a timely exemplar to discuss the barriers, enablers and incentives needed to find effective treatments in advance of health emergencies caused by emerging infectious diseases.

This article is part of the themed issue ‘The 2013–2016 West African Ebola epidemic: data, decision-making and disease control’.

## 1. Introduction

The 2013–2016 Ebola virus disease (EVD) outbreak in West Africa exposed many failings in the international community's ability to respond swiftly and effectively to an epidemic of an emerging infectious disease. One important aspect was the lack of licenced treatments available at the start of the epidemic, despite almost 40 years of Ebola outbreaks.

An effective therapy for EVD is important for a number of reasons. With historical case fatality rates as high as 89% [1], the humanitarian imperative to offer better care is clear. Furthermore, access to treatment may incentivise patients to seek care and inspire confidence in the intentions of healthcare workers. Improving survival is also important for public health control of the epidemic—modelling for the most recent EVD outbreak indicated that an effective therapy would reduce the total number of cases independently of other methods of control [2]. Minimizing case numbers also makes economic sense; the West Africa EVD epidemic slowed economic growth and led to an increase in poverty by an estimated 7–16% in the most affected countries [3].

The failure to provide effective treatments for EVD is destined to be repeated for other epidemic-prone infections unless there are improvements in the ways therapeutics are developed and evaluated. Indeed, progress for some key emerging infectious diseases has been poor. There have been 1733 laboratory confirmed cases of Middle East respiratory syndrome coronavirus (MERS-CoV) from September 2012 to 20 June 2016 [4], but pre-clinical therapeutic development has been insufficient to advance to human trials [5]. Only two prospective interventional trials were published during the most recent influenza (H1N1, 2009) pandemic, an outbreak that infected approximately 20–27% of the population in some affected regions [6], and likely caused tens of millions of cases worldwide [7].

During the West Africa EVD epidemic however, efforts to progress pre-clinical development and to overcome regulatory, ethical and operational challenges to conduct patient centred research during the outbreak were successful. This included the first human clinical trials in patients with EVD. This piece, therefore, will discuss areas in which this progress can be extended to meet the critical need for treatments for other emerging infectious diseases.

## 2. Part 1: A stocktake of achievements—pre-clinical development of therapeutics for Ebola virus disease

Various therapeutic agents were under pre-clinical development for EVD prior to the outbreak. Table 1 outlines the most promising candidate therapies and their development status at the beginning of 2014. This work was no doubt facilitated by national security concerns—EVD is one of the few diseases in the highest priority (A) category for national security and public health threat determined by the National Institute of Allergy and Infectious Diseases (NIAID) biodefense research disease programme [17] and all of the most developed candidate drugs were advanced with funding from military agencies. As an example, the US Defense Threat Reduction Agency (DTRA) allocated 300 million USD for medical countermeasures for viral haemorrhagic fevers in the decade before the outbreak [18].

**Table 1.** Financing of promising candidate EVD therapeutics, prior to 2013–2016 West Africa outbreak. Data courtesy of Open Society Foundation (2014, unpublished report: Overview of intellectual property claims and public research support for candidate Ebola vaccines and therapies). CRADA: Cooperative Research and Development Agreement; US: United States of America; DTRA: Defense Threat Reduction Agency, US; USAMRIID: The United States Army Medical Research Institute for Infectious Diseases; NIAID: National Institute of Allergy and Infectious Diseases, US; DOD: Department of Defense; NIH: National Institute of Health, US; BARDA: Biomedical Advanced Research and Development Authority, US; CMV, cytomegalovirus.

			disbursed or committed public funding		
candidate drug	initial target	year of first patent filing	amount (USD)	source	pipeline development at outbreak start
brincidofovir	smallpox and DNA viruses (CMV)	2004	36.1 million	US NIH [8]	*other applications*: smallpox—unknown *EVD*: none
			CRADA, value unknown	USAMRIID	
			53 million	BARDA	
			5 million	NIH	
favipiravir	influenza	1998	138.5 million	DTRA	*other applications*: pandemic influenza—licenced for stockpiling (Japan) [9] *EVD*: 100% survival in mouse model [10]
TKM-Ebola	target specific to EVD, but delivered using technology applicable to other diseases	2005	44 million	US DOD	*other applications*: n.a. *EVD*: 100% survival in non-human primate model [11]
			26 million as part of a consortium	NIH	
BCX4430	hepatitis C, yellow fever	2005^a^	20 million	NIAID	*other applications*: yellow fever—100% survival in hamster model [12]. *EVD*: none
AVI-7537	target specific to EVD, closely related to precursor drug for Marburg disease	2010	28 million	DTRA	*other applications*: close relative in phase I trials for Marburg virus [13] *EVD*: support terminated by US Government [14]
			11 million	US defense appropriations earmark	
			<80 million	US DOD	
JK-05	unknown; ‘a biodefence drug’	2010^b^	unknown	Peoples Liberation Army, China	*other applications*: unknown *EVD*: unknown, no human trials
ZMapp (and precursors ZMab and MB-003)	EVD	2008	189 000	US army	*other applications*: n.a. *EVD*: 100% survival in non-human primate model [15]
			24.9 million	NIAID	
			5.2 million	DTRA	
			2.6 million	DTRA	
			CRADA, amount unknown [16]	Public Health Canada	

^a^With some uncertainty (multiple components).

^b^With some uncertainty (insufficient information available).

However, the pipeline was far from robust and pre-clinical progress had stalled for some agents despite promising preliminary data. For example, a registered phase I human clinical trial of the antiviral AVI-7537—which would have been the first human clinical trial of an EVD therapeutic—was terminated in 2012 prior to enrolment due to government funding restraints [14]. Likewise, despite non-human primate trials demonstrating 100% efficacy for TKM-100802 and ZMapp [11,15], these results had not yet been published at the time the West Africa outbreak started, and phase I human trial results were also not publicly available.

Pre-clinical progress accelerated as the epidemic progressed. In 2014, at least 70 million USD was spent on EVD drug development [19]. Some of this was pipeline progression on existing candidates, including a number of new animal studies. A surge in compound library screening studies also attempted to identify existing FDA approved agents for repurposing. However, the yield from this activity was low within the timeframe of the epidemic, with the exception of brincidofovir (that progressed to a clinical trial).

### (a) An improved paradigm: new models for supporting research and development of new therapeutics

The lack of an available treatment for EVD, or at least a drug that was ready to go straight into patients enrolled in a clinical trial, at the beginning of the epidemic was a significant failure. An efficient, robust drug development pipeline that brings promising agents to human clinical trials before an outbreak should be viewed as an integral component of global health preparedness. There is clear economic sense for governments in doing so—the average annualized economic loss from pandemics is estimated at 60 billion USD [20]. As an example, the 2003 SARS outbreak that resulted in 800 deaths cost an estimated 54 billion USD, primarily carried by the public sector [21]. However, pre-clinical drug development for rare and poverty related diseases is characterized by market failures with private sector research and development (R&D) priorities being based on profit prospects from patent monopolies and large margins rather than health needs [22,23]. While useful for rewarding companies for developing medicines for wealthy health systems and markets, these incentive mechanisms are ineffective when there is a low-profit prospect (either small patient numbers or low purchasing power of the affected population) or an unpredictable market (such as in the case of an epidemic). Unfortunately, increasingly seen as a tool to promote economic growth, public R&D funding for pharmaceuticals has followed private sector priorities and is made contingent to partnerships with the industry, and disproportionately targets issues relevant to high income countries [23]. However, given the high cost of not being able to control an outbreak in a timely way, there is a case to be made for increased public investment to secure a robust pre-clinical candidates pipeline, ready to be tested in patients in case of an outbreak.

Aimed at remediating the failure of the current R&D model to prioritize health needs and deliver affordable products, a variety of reports have been released that suggest ways to strengthen global financing for R&D for neglected and epidemic diseases and ensure affordable access [21–23]. A recurrent suggestion is to better lever the existing public sector funding already spent on drug development, in particular by ensuring that the resulting products are affordable and accessible [22]. For example, during the 2014 financial year, it is estimated that 90% (64 million USD) of EVD drug research funding was provided by the public sector [19], but it is unclear to what extent product accessibility and affordability were negotiated as a condition of this funding. There is also general agreement that alternative incentive mechanisms are required to replace drug patenting, but there is less consensus on which mechanisms to promote. Common suggestions include market push and pull mechanisms such as increased public research funding or advanced purchase agreements, or more innovative mechanisms such as the use of prizes (for progress or completion) and other ways that uncouple paying for R&D and product sales (‘delinkage’). Not-for-profit Product Development Partnerships (PDPs) involving public and private sectors such as the Medicines for Malaria Venture (MMV, www.mmv.org) or the Drugs for Neglected Diseases initiative (DNDi, www.dndi.org) have demonstrated their effectiveness for specific neglected disease niches.

Furthermore, it is important for these incentive mechanisms to be efficient, that they do not occur in silos. It is clear that the research response to EVD was slowed by ‘insufficient collaboration and transparency’ [21]. There was wasted effort from duplication of screening of compounds that had already been deemed unlikely drug candidates in other studies. Furthermore, the World Health Organization (WHO) were receiving ‘almost daily’ proposals for potential EVD treatments where there were already data indicating many of these agents were ineffective against the virus [24]. This problem is compounded by proprietary data held by commercial companies, and some secrecy around research funded through military channels. The development of collaborative frameworks that can pool R&D funding towards priority health goals, mobilize the appropriate public and private partners, and ensure affordability and accessibility for the affected population are desperately needed.

A possible solution exists in adopting the new collaborative models developed for vaccine development. For example, the Coalition for Epidemic Preparedness Innovations (CEPI; www.cepi.net) provides one such initiative, aimed at approaching gaps in the vaccine pipeline for emerging diseases, focusing on diseases where market failure currently exists. This initiative is significant for several reasons. Firstly, the diversity of representation in the founders (including the Gates Foundation, India's Department of Biotechnology, and the World Economic Forum) and likewise in the organization's board, should help prevent unnecessary duplication of effort by producing a unified strategic direction, with secure funding. Collaboration between different sectors should also improve the quality of horizon scanning, so that efforts and financing are directed toward the most likely threats. The end-to-end leadership possible in such a large consortium may have significant impact in providing a platform for rapid acceleration of vaccine development through all stages of the pipeline when a novel threat is encountered. It is arguable that such a model could be adopted for pre-clinical drug development given the vast similarities in the problem being addressed. It is also worth considering to what extent aspects of this ambitious approach can be used for supportive care strategies, and how elements can be adopted to clinical trial networks.

## 3. Part 2: A stocktake of achievements—clinical trials of therapeutics for Ebola virus disease

Despite outbreaks occurring over a period of four decades, no clinical trials of an EVD therapeutic had ever been conducted in humans before 2014. However, convalescent blood had been administered on a compassionate basis during the 1995 Kikwit outbreak (with unclear efficacy) [25].

During the 2013–6 epidemic, promising experimental therapies were first administered under a compassionate basis to healthcare workers who were being treated in high income settings [26]. Viewed by some as a further manifestation of the unethical discrepancies in care being provided to those affected in West Africa and others elsewhere, these events fuelled a growing number of calls for experimental agents to be made available to patients in the most affected region. In September 2014, shortly following the announcement of the Public Health Emergency of International Concern (PHEIC), WHO convened an expert committee consultation to determine if and how therapeutic testing should progress. They affirmed the findings of an earlier ethics panel that there was a moral imperative to evaluate these treatments, within an umbrella of safe and ethical conduct, and under the condition that results were made available to further knowledge of the disease [27]. WHO also noted the extraordinary circumstances of the epidemic and sanctioned ‘innovative methods of rapid assessment’ to help identify safe and effective countermeasures quickly [28]. Shortly after, several funding agencies committed to funding therapeutic trials. Again, these were predominantly from the public sector and included the Wellcome Trust, National Institutes of Health and the European Commission.

Subsequently, patients began receiving experimental therapies in the most affected regions. The first use of an experimental agent appears to be provision of convalescent blood in December 2014, led by the staff at the 34 Military hospital in Freetown, Sierra Leone, although details of this use have not been published. The first registered therapeutic clinical trial (favipiravir) did not begin until 17 December 2014, by which time, over 18 600 cases had already been reported to WHO [29]. Overall, seven trials opened to patient recruitment [30] and fifteen different therapeutics have been used in humans [31]. Several main approaches were taken. Investigation of convalescent blood and blood products (plasma) were the initial preference of WHO, in the context of clear safety data and scalability. Novel (TKM-130803) and repurposed (favipiravir) antivirals were also trialled and several monoclonal antibody cocktails were used (although only ZMapp in a clinical trial setting). Following the acute phase of the epidemic, a clinical trial of the investigational therapy GS-5734 has recently (June 2016) been announced to assess Ebola virus eradication in the semen of survivors (clinical trial identifier NCT02818582). Trials of other repurposed agents (including azithromycin, sunitinib, erlotinib, atorvastatin, irbesartan and amiodarone) were planned but did not recruit patients and assessment of other agents (including amiodarone, atorvastatin + irbesartan (+/− clomiphene), lamivudine) provided insufficient details to WHO to make an assessment of efficacy [32]. Almost all of the registered clinical trials were conducted in partnerships between investigators in the most affected countries and international experts. Unusually, in many cases these trials were also conducted with non-governmental organization (NGO) partners that were providing patient care, but who would not normally participate in drug development trials [33].

Three clinical trials have published their results. The Ebola Tx convalescent plasma trial demonstrated no overall survival benefit, although data on neutralizing antibody titres in this cohort are needed to determine if more targeted approach holds promise in the future [34]. A trial of TKM-130803 conducted in Sierra Leone found no survival benefit compared with historical controls [35]. Investigation of favipiravir determined that it was unlikely to be of benefit to patients with high viral load, but that further investigation is warranted in patients with less pronounced viraemia [36]. In addition, interim reporting of the ZMapp trial indicates no conclusion to date, but some promise [37].

### (a) An improved paradigm: explore, test and implement a suite of trial design options

Early during their assessment of research priorities for the outbreak, WHO announced support for alternative trial designs. The reasons for this included concerns around the ethics and acceptability of randomization in the setting of high mortality and community distrust—concerns voiced by some scientists and advocates from the most affected countries [27,38]. Furthermore, it was hoped that the potentially smaller number of patients needed to reach statistical conclusions under certain conditions in these trial designs would help triage treatments faster [39]. As a result, single arm and adaptive design trials commenced, followed at a later stage by randomized controlled trials. Although there was dispute among scientists as to the most appropriate trial designs in this setting, there was most certainly innovation in the approaches taken, and this needs to be used as a springboard for further advances. Several areas of planning are possible to advance now, with the aim of having off-the-shelf, operationally tested trial designs available at the onset of cases.

**Figure 1.**
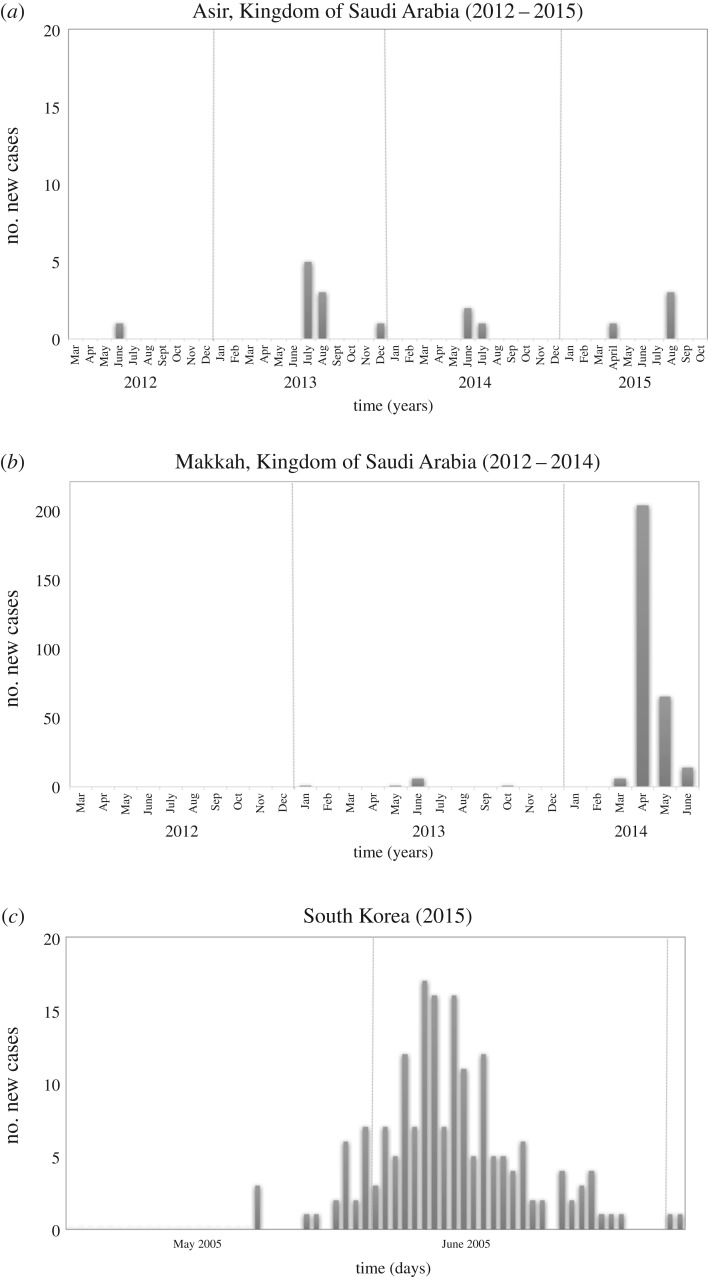
Possible epidemiological patterns for diseases with epidemic potential, as demonstrated by MERS Co-V cases in three different regions. In each of these regions, the possible number of patients eligible for recruitment and the time in which to enrol them differ markedly, despite the same causative pathogen: (*a*) small intermittent outbreaks over time, (*b*) small intermittent outbreaks followed (at marked time point) by a propagated outbreak and (*c*) propagated outbreak. Data adapted from that publicly available at https://public.tableau.com/profile/ian.m.mackay#!/vizhome/MERS-CoV_0/.

Firstly, while modelling of epidemic threats are often undertaken for public health reasons, to date, very little work has used predictable elements of outbreak epidemiology to investigate the feasibility of different trial designs. In figure 1, using MERS-CoV as an example, we outline three likely epidemiological scenarios for emerging infections that even in simplistic form can help identify potential issues for trial design. For example, it is evident, but rarely made explicit, that for some emerging infectious diseases that result in a small number of sporadic cases with uncertain timing and location (figure 1*a*), there can be no certainty that enough cases will accrue to complete an hypothesis driven randomized controlled trial. The issue of unpredictable numbers and difficulty in recruitment has been faced in other fields of clinical research, particularly research on rare inherited diseases and rare cancers, but it is also likely to become an increasing problem in studies of uncommon phenotypes of antimicrobial resistance [40,41]. However, with infectious diseases there is always the possibility that a larger outbreak may cause a spike in case numbers (figure 1*b*). For such a scenario adaptive Bayesian trial designs may be a solution, where, while numbers of cases are small, the initial focus is on estimating the treatment effect and safety rather than hypothesis testing, and drug registration may be possible with supplementary data from animal models. Such a trial could be designed so that in the event of a substantial outbreak the trial could switch to hypothesis testing while using the data already accrued. For larger outbreaks (figure 1*c*), adaptive ‘platform trials’ may be an efficient option, which allow the testing of multiple agents simultaneously, even if there is no agreed best comparator group [42,43].

Of course, not all elements of a trial design can be designed in advance. The most significant limitations include the evolving understanding of the natural history of an emerging disease, and that the research priorities of the affected populations (who should be prioritizing trials) may differ depending on the location and circumstances of the outbreak.

### (b) An improved paradigm: between outbreaks accrue evidence in analogous infections

Now that the epidemic has concluded, there is a risk that the forward momentum of EVD research wanes. This is especially the case for patient-centred research that necessarily relies on individuals with the disease to test hypotheses. It is also possible that a full-scale trial may not be feasible if the next outbreak is small. A consideration for enabling progress is to advance knowledge in similar but more common infections and supplement this with smaller bridging studies that demonstrate that extrapolation to the emerging infectious disease is valid.

This approach has been suggested for rare antimicrobial resistance phenotypes [44]. Such an approach would for example be ideal for progressing the clinical development of drugs for avian influenza viruses, where proof of principle in seasonal influenza can then be bridged to rarer human cases of avian influenza infection. Between 3 and 5 million people are severely ill due to seasonal influenza epidemics each year [45]. Despite this, there are relatively few large therapeutic trials with a clinically significant primary outcome (death, ICU admission), and even fewer for high risk populations such as pregnant women. Given the significant similarities in disease pathogenesis, drug target, and at risk populations between seasonal and pandemic influenza, this is a wasted opportunity to not only improve seasonal influenza care, but also to produce data driven statistical designs and endpoints for trials of novel influenza strains causing outbreaks, and extendable trial platforms.

This approach would be particularly suited to studies of host-directed therapies and trials of supportive care interventions, where common pathological pathways are targeted. For example, during the West Africa EVD outbreak, the experimental agent FX06 was provided on a compassionate care basis to two patients medically evacuated to high income settings (although not used in a clinical trial) [46]. FX06 is a host-directed therapy aimed at reduction of vascular instability and capillary leak. The drug had demonstrated encouraging results in a mouse model of dengue virus-induced shock syndrome (DVISS) [47] and it was based on the purported resemblance of the vascular leak in this syndrome with that of EVD that patients were provided the drug. While the extent of pathophysiological similarity between the two diseases is not well characterized, progression of the drug to clinical trials for DVISS or other diseases causing vascular damage will provide valuable information, for example regarding pharmacokinetics, for better clinical trials during an outbreak.

There are a number of benefits to adopting this groundwork by analogy approach. Firstly, similarities in the dosing and toxicity profile of the drug between diseases may help produce effective trial protocols if and when emergency trials are implemented. In addition, operational capacity is enhanced in centres recruiting patients during ‘peace time’ who can then respond more adequately during an outbreak. This is especially important if the opportunity is taken to conduct clinical trials in resource-poor settings where epidemic potential is high. In this scenario, capacity building enables local investigator leadership during an outbreak. However, there are limits to the extent that extrapolation is justified, safe and ethical and these require thorough examination each time this approach is considered.

### (c) An improved paradigm: consider supportive care when prioritizing trials

Improvements in supportive care probably contributed to the moderate gains in survival over time during the West Africa epidemic. However, the safety and efficacy of individual components remains unknown because no trials were conducted. In particular, there remains contention regarding optimal fluid resuscitation strategy, use of empiric antimicrobials, anti-diarrhoeals, non-steroidal anti-inflammatory agents and vitamin K. Many of these questions are applicable to other viral haemorrhagic fevers in the least, but probably also to other emerging infectious diseases—because there is limited understanding of the pathophysiology of these conditions, and also due to the poor representation of patients from high risk countries in existing medical literature. Unexpected findings of potential harm with fluid bolus provision in critically ill children [48] and adults with sepsis [49] in resource limited settings demonstrate the risk of unexamined implementation of ‘usual care’ principles from high resource settings. There are several key benefits to prioritization of supportive trial therapies. Firstly, significant gains in patient survival may be possible without the ‘silver bullet’ of an effective novel agent. Gains in sepsis survival over the last few decades provide an excellent exemplar given the lack of specific therapy for that syndrome despite a large volume of research. Secondly, these trials are not reliant on a costly and time-consuming drug development pathway to be completed and do not suffer the issues of post-trial pricing and accessibility to the same extent as novel therapies—it should be more operationally feasible to implement findings into limited resource settings. However, given there is often a limited number of appropriate trial sites during an outbreak, crucial considerations include deciding what factors (such as predicted benefit of a drug, accessibility) should determine how supportive care trials are prioritized relative to novel drug trials, or under which circumstances simultaneous evaluation of supportive and interventional measures can occur.

### (d) An improved paradigm: prioritizing clinical trials for emerging and other infections

That several clinical trials were launched during the timeframe of the EVD epidemic is undoubtedly a success. However, this, perhaps for the first time, led to the unintended consequence of multiple trials vying for enrolment during the final phases of the epidemic, as case numbers fell. Allegations of ‘chaotic land grabs for sites and patients' surfaced [50], despite good collaboration between many research groups.

There are multiple consequences of too many clinical trials enrolling simultaneously. Most importantly, a fractured research response can lead to inadequate sample size enrolment in each trial and an overall failure to identify drugs that improve patient survival, or that do not work. There is the risk of overwhelming regulatory authorities in the affected region who are required to deal with a surge in applications to conduct trials. For example, the number of applications to the national ethics committee in Guinea increased fourfold in 2014–15 compared with the previous year [51]. During the EVD epidemic there were very few senior, research experienced clinicians from the affected countries available to lead trials and many of these also had significant responsibility in the humanitarian response to the emergency. Subsequently, overlap of principal investigators between trials was substantial and they risked being placed in a compromised position due to the competing interests of different groups. For future outbreaks, it is worth considering triaging of clinical trials, and potential clinical trial locations. This is a decision that should be led by the most affected countries, but WHO are likely required to convene all appropriate stakeholders.

If appropriate prioritization of therapeutic interventions and/or trial designs can occur in advance of an outbreak, there is another important benefit. Of the delays to clinical trials during the West Africa EVD epidemic, the weeks to months taken to develop and authorize partnership contracts were especially needless—it is entirely feasible for components of these contracts to be produced in advance. These contracts delineate the legal and financial responsibilities of the trial partners and during the epidemic these were developed *ad hoc*, as trial site access was negotiated. However, the international epidemic diseases research consortiums and networks that are likely to provide expertise during an epidemic are already known. Likewise, the non-governmental agencies that will be engaged in a research response in a given region are foreseeable. As mapping of medical countermeasure pipelines improves for epidemic diseases, the companies and research institutions involved with the most promising candidates can be engaged early and ownership issues including possible intellectual property clarified. As there are predictable areas of negotiation (e.g. post-trial drug pricing and access conditions, reflecting the respective public and private investments) for each of these stakeholders, pre-prepared contracts would be an excellent outcome of increased collaboration.

The most significant difficulty in generating ‘peace time’ protocols is ensuring that local governments and populations are adequately represented when the location of the next epidemic is not known. In the quest for a harmonized, rapid research response, it is important that globally agreed priorities do not discount the specific needs and agenda of local stakeholders. Early recognition of the most appropriate local representatives during an outbreak is therefore critical, but often can be hindered by poor research infrastructure or unclear government organization.

## 4. Conclusion

For far too long, an inadequate R&D pipeline has been a feature of disease outbreaks caused by emerging and epidemic infections. Strategic efforts for epidemic prevention and control must take a more innovative approach to securing the R&D pipeline for promising treatments and accelerating the conduct of rapid, flexible clinical trials. Potential solutions include the creation of ambitious, multi-sector initiatives for pre-clinical development and improved use of inter-epidemic periods to progress clinical trials through analogous diseases, conduct methodological work on trial design, and triage trials in preparation for the next outbreak. Most critically, all these initiatives require a renewed commitment to collaboration—by funders and researchers—that prioritizes the needs of patients and communities, and reduces unnecessary duplication and delays.
